# Enzyme Activity in Relation to Cancer

**DOI:** 10.1038/bjc.1957.71

**Published:** 1957-12

**Authors:** E. Boyland, D. M. Wallace, D. C. Williams


### ENZYME ACTIVITY IN RELATION TO CANCER

### INHIBITION OF URINARY $\beta$-GLUCURONIDASE OF PATIENTS WITH CANCER OF THE BLADDER BY ORAL ADMINISTRATION OF 1 : 4-SACCHAROLACTONE AND RELATED COMPOUNDS

E. BOYLAND, D. M. WALLACE AND D. C. WILLIAMS

*From the Chester Beatty Research Institute, Institute of Cancer Research: Royal Cancer Hospital, London, S.W.3; The Royal Marsden Hospital, London, S.W.3 and the Institute of Urology, London, W.C.2*

Received for publication September 20, 1957

A NUMBER of clinical and statistical observations led to the suggestion (Boyland, Wallace and Williams, 1955) that patients suffering from cancer of the bladder excrete carcinogenic substances in the urine. The investigation of Bonser, Bradshaw, Clayson and Jull (1956) and of Allen, Boyland, Dukes, Horning and Watson (1957) indicate that a number of *ortho*-aminophenols can induce cancer in experimenta lanimals. Two *ortho*-aminophenols, both metabolites of tryptophan, are nórmally present in urine and these substances, 3-hydroxyanthranilic acid and 3-hydroxykynurenine, have produced tumours when implanted in the bladders of mice (Allen *et al*, 1957). Both 3-hydroxyanthranilic acid and 3-hydroxykynure-

FIG. 1.—The equilibrium between naturally occurring *ortho*-aminophenols and the corresponding detoxication products.

nine were shown to be present in increased amounts in the urine of patients suffering from cancer of the bladder (Boyland and Williams, 1954, 1956). *ortho* -Aminophenols are usually excreted as sulphuric esters or as glucuronides in urine and these compounds would be expected to be inactive as carcinogens unless hydrolysed by enzymes. The carcinogen concentration would be dependent on the time which the urine remained in the bladder, on the concentration of *ortho*-aminophenol conjugates in the urine and on the urinary activity of the enzymes sulphatase and $\beta$-glucuronidase (Fig. 1). The activity of both sulphatase and $\beta$-glucuronidase in urine is raised in cancer of the bladder (Boyland, Wallace and Williams, 1955) but urinary sulphatase hydrolyses the sulphuric esters of some carcinogenic aminophenols only slowly (Boyland, Manson, Sims and Williams, 1956) and therefore β-glucuronidase must play a part in the hydrolysis of such conjugates. If the urinary β-glucuronidase activity in patients, in which new tumours would be expected to develop, could be inhibited, then these tumours should be prevented.

The present work deals with the urinary β-glucuronidase activity of patients suffering from cancer of the bladder and the effect of treatment with β-glucuronidase inhibitors on the enzyme activity. The inhibitors used are all derived from glucosaccharic acid, which have been previously shown to inhibit β-glucuronidase *in vitro* (Karunairatnam and Levvy, 1949; Mills and Paul, 1949; and Levvy, 1952). Saccharo-1 : 4-lactone (glucosaccharo-1 : 4-lactone) is the most effective inhibitor and the inhibitory action of saccharates used in these investigations is probably due to the 1 : 4-saccharolactone which they contain or is produced from saccharate in the body. Saccharic acid is readily and reversibly converted to a mixture of saccharo-1 : 4-lactone and saccharo-3 : 6-lactone.

Saccharo-1 : 4-lactone (II) is formed from saccharic acid (III) by loss of the elements of water. This dehydration reaction which is reversible gives the lactone (II) which has a structural resemblance to glucosiduronic acids (*cf.* I). Glucosaccharo-1 : 4-lactone is probably an active inhibitor of β-glucuronidase because of this similarity in structure which allows it to occupy the enzyme centre which is concerned with β-glucuronidase activity. Glucosaccharo-3 : 6-lactone (IV) which is also formed from saccharic acid and only differs from the 1 : 4-lactone, in the position of hydroxyl groups is a poor inhibitor. (For formulae I to IV see page 585).

### EXPERIMENTAL

The β-glucuronidase activity is almost always increased in the urine of patients with cancer of the bladder and remains high in most cases even after the tumour has been removed (Boyland, Wallace and Williams, 1955). The method of estimation has since been modified (Boyland *et al.*, 1957) in the following ways—(*a*) A 20 per cent solution of thymol in benzene is used as a preservative as it is more effective than the benzene used in the original work. (*b*) The buffer concentration has been increased so that the activity of alkaline urine specimens (previously discarded) may also be estimated. The enzyme is unstable in strongly alkaline solution. The upper limit of the normal range using this method is 1·2 units/ml. All β-glucuronidase values are expressed as units of activity per ml. of urine. The unit of activity is defined as being that which liberates 1 μg. of phenolphthalein per hour at 37° C. In all cases the total 24 hr. output of urine was collected and the test performed on a 1 ml. aliquot of this; the total 24 hr. volume being also quoted so that the total 24 hr. excretion may be calculated. All patients included have a history of more than 2 years of cancer.

### *The treatment of patients with enzyme inhibitors*

Patients have been treated with a number of inhibitors (supplied by Messrs. Kemball, Bishop and Co. Ltd., London, E.3) to decrease the β-glucuronidase activity in urine. The estimation of urinary β-glucuronidase involves a 3-fold dilution so that the apparent inhibition obtained is always less than the actual effect in the urine. Patients were treated with solutions of the inhibitors flavoured with " fruit concentrates ".

(a) *Calcium gluconate.*—The average inhibition of urinary $\beta$-glucuronidase activity on treatment with 10 g. calcium gluconate is only 15 per cent (Table I).

TABLE I.—*Patients Suffering from Cancer of the Bladder Treated with Calcium Gluconate*

| | | | Before treatment | | During treatment | |
|---|---|---|---|---|---|---|
| Case No. | Sex | Age | Vol./24 hr. | Units/ml. | Vol./24 hr. | Units/ml. |
| 1 | M. | 59 | 2750 | 1·8 | 2700 | 1·2 |
| | | | 2750 | 1·6 | 2500 | 0·8 |
| 2 | ,, | 63 | 1400 | 5·8 | 2750 | 3·8 |
| | | | 2350 | 5·5 | 2750 | 5·8 |
| | | | 4000 | 5·8 | — | — |
| 3 | ,, | 77 | 1800 | 1·7 | 2400 | 1·7 |
| | | | 2000 | 1·9 | 2850 | 1·8 |
| | | | 1950 | 2·0 | 2850 | 1·6 |
| 4 | ,, | 67 | 2750 | 1·5 | 3100 | 1·4 |
| | | | 3500 | 1·8 | 2700 | 1·3 |
| | | | 2750 | 1·2 | 3700 | 1·4 |
| 5 | ,, | 65 | 2900 | 1·9 | 3100 | 1·6 |
| | | | 3100 | 2·1 | 2500 | 1·3 |

(b) *Sodium ammonium saccharate.*—A patient with multiple tumours of the bladder was treated with sodium ammonium saccharate solution (10 g. per day for 1 week) (Fig. 2). The treatment was then stopped and the $\beta$-glucuronidase activity rose to the original value within 3 days. The variation in enzyme activity and the volume of the 24 hr. urine specimen is also recorded. The results obtained from 4 patients treated in a similar manner (Table II) indicate that the treatment reduced the $\beta$-glucuronidase activity by 31 per cent.

TABLE II.—*Patients Suffering from Cancer of the Bladder Treated with Sodium Ammonium Saccharate*

| | | | Before treatment | | During treatment | |
|---|---|---|---|---|---|---|
| Case No. | Sex | Age | Vol./24 hr. | Units/ml. | Vol./24 hr. | Units/ml. |
| 1 | M. | 52 | 3700 | 3·2 | 3100 | 1·2 |
| | | | 4000 | 2·7 | 3500 | 1·7 |
| | | | 3100 | 2·6 | 3900 | 1·2 |
| 2 | ,, | 75 | 1700 | 3·0 | 3300 | 2·7 |
| | | | 2000 | 2·3 | 2800 | 2·0 |
| | | | 1800 | 2·3 | — | — |
| 3 | ,, | 62 | 2500 | 1·6 | 1900 | 1·1 |
| | | | 4300 | 1·3 | 2100 | 1·0 |
| | | | 2700 | 1·7 | — | — |
| 4 | ,, | 38 | 8000 | 0·8 | 3300 | 1·1 |
| | | | 6700 | 1·2 | 3600 | 0·7 |
| | | | 9250 | 1·2 | 3500 | 0·9 |
| | | | 7300 | 1·0 | 4400 | 1·0 |

FIG. 2.—The effect of oral administration of sodium ammonium saccharate on urinary β-glucuronidase activity in a patient with bladder cancer.

○——○ β-Glucuronidase activity (units/ml.).
●......● Urine volume (litres/24 hr.).

FIG. 3.—The effect of oral administration of ammonium hydrogen saccharate on urinary β-glucuronidase activity in a patient with bladder cancer.

○——○ β-Glucuronidase activity (units/ml.).
●——● Urine volume (litres/24 hr.).

(c) *Ammonium hydrogen saccharate.*—Treatment of a patient with 10 g. per day of ammonium hydrogen saccharate (Fig. 3) produced a fall in β-glucuronidase activity of the urine. Treating 6 patients with 3·3 g. of ammonium hydrogen saccharate 3 times daily produced an average apparent inhibition of the β-glucuronidase activity of 47 per cent (Table III).

TABLE III.—*Patients Suffering from Cancer of the Bladder Treated with Ammonium Hydrogen Saccharate*

| | | | Before treatment | | During treatment | |
|---|---|---|---|---|---|---|
| Case No. | Sex | Age | Vol./24 hr. | Units/ml. | Vol./24 hr. | Units/ml. |
| 1 | F. | 51 | 2700 | 1·4 | 2750 | 0·65 |
| | | | 2850 | 1·8 | 2810 | 0·80 |
| | | | 2750 | 1·8 | 2520 | 0·70 |
| | | | 3250 | 1·6 | 2750 | 0·65 |
| | | | 2520 | 1·7 | 2450 | 0·80 |
| 2 | M. | 54 | 3100 | 0·50 | 2800 | 0·30 |
| | | | 2850 | 0·65 | 2700 | 0·35 |
| 3 | ,, | 47 | 3600 | 1·8 | 2000 | 1·2 |
| | | | 2500 | 1·4 | 2250 | 1·0 |
| 4 | ,, | 61 | 1800 | 1·6 | 2100 | 0·60 |
| | | | 1650 | 1·7 | 2200 | 0·65 |
| | | | 2750 | 1·6 | 2600 | 0·80 |
| 5 | ,, | 56 | 700 | 0·55 | 1600 | 1·0 |
| | | | 700 | 0·92 | — | — |
| 6 | F. | 56 | 800 | 2·6 | 2100 | 1·25 |
| | | | 2000 | 3·6 | 2600 | 1·1 |
| | | | 2000 | 1·9 | — | — |

(d) *Glucosaccharolactone.*—(1) A group of patients were treated with a boiled solution of saccharic acid which contains an equilibrium mixture of saccharic acid and 3 : 6 and 1 : 4 saccharolactones, and received 1·3 g. of saccharo-1 : 4-lactone 3 times daily. The effect of this treatment on the urinary β-glucuronidase activity (Fig. 4 and Table IV) indicates that the mean inhibition of β-glucuronidase activity is 92 per cent.

TABLE IV.—*Patients Suffering from Cancer of the Bladder Treated with Boiled Saccharic Acid Solution*

| | | | Before treatment | | During treatment | |
|---|---|---|---|---|---|---|
| Case No. | Sex | Age | Vol./24 hr. | Units/ml. | Vol./24 hr. | Units/ml. |
| 1 | M. | 54 | 2000 | 0·90 | 2400 | 0·01 |
| | | | 1900 | 1·3 | 3100 | 0·03 |
| | | | 2600 | 1·4 | 3300 | 0·02 |
| | | | 1600 | 1·0 | 2900 | 0·00 |
| 2 | F. | 38 | 2500 | 1·4 | 2500 | 0·35 |
| | | | 2650 | 0·92 | 2610 | 0·21 |
| | | | 2520 | 1·6 | 2120 | 0·14 |
| | | | 2760 | 1·0 | 1800 | 0·07 |
| 3 | ,, | 55 | 2550 | 1·6 | 1100 | 0·44 |
| | | | 1750 | 1·0 | 2500 | 0·06 |
| 4 | M. | 48 | 980 | 3·6 | 1010 | 0·10 |
| | | | 910 | 2·7 | 1100 | 0·05 |
| 5 | ,, | 34 | 3060 | 1·22 | 4050 | 0·08 |
| | | | 4460 | 0·96 | 3700 | 0·10 |

FIG. 4.—The effect of oral administration of boiled saccharic acid on urinary β-glucuronidase activity in a patient with bladder cancer.

○———○ β-Glucuronidase activity (units/ml.).
●......● Urine volume (litres/24 hr.).

(2) Treatment of a number of patients with a mixture of glucosaccharo-1 : 4-lactone and glucosaccharo-3 : 6-lactone (2·5 g. of the mixture containing 1 g. of the 1 : 4-lactone administered 4 times daily) (Table V) caused an average inhibition of activity of 84 per cent.

TABLE V.—*Patients Suffering from Cancer of the Bladder Treated with a Mixture of Glucosaccharo*-1 : 4-*lactone and Glucosaccharo*-3 : 6-*lactone*

| Case No. | Sex | Age | Before treatment Vol./24 hr. | Before treatment Units/ml. | During treatment Vol./24 hr. | During treatment Units/ml. |
|---|---|---|---|---|---|---|
| 1 | F. | — | 1825 | 4·7 | 2210 | 1·6 |
| | | | 1550 | 10·3 | 2140 | 1·2 |
| 2 | ,, | 76 | 1595 | 7·6 | 1865 | 1.3 |
| | | | 1535 | 6·1 | — | — |
| | | | 1335 | 6·1 | — | — |
| 3 | M. | 66 | 2640 | 6·3 | 1940 | 1·7 |
| | | | 2850 | 7·3 | 1725 | 0·28 |
| | | | 2550 | 6·2 | 1585 | 0·56 |
| 4 | ,, | 61 | 2700 | 2·8 | 2610 | 0·33 |
| | | | 2100 | 3·1 | 2490 | 0·33 |
| 5 | ,, | 82 | 1510 | 4·9 | 2100 | 0·71 |
| | | | 1575 | 3·2 | 1765 | 0·43 |

(3) The results obtained by treatment of patients with pure saccharo-1 : 4-lactone (1 g. 4 times daily) (Table VI) show a mean inhibition of 93 per cent. The effect of administration of both pure saccharo-1 : 4-lactone and of the 1 :4,

3 : 6 mixture on the urinary β-glucuronidase of patients suffering from cancer of the bladder is shown in Fig. 5. Specimens of urine were collected from each of 5 patients at approximately 3 hourly intervals and the β-glucuronidase activity of the individual specimens estimated. In 5 successive periods each of 48 hr. the

FIG. 5.—The effect of oral administration of glucosaccharo-1 : 4-lactone and mixtures of 1 : 4 and 3 : 6-lactones on urinary β-glucuronidase activity in 5 patients with bladder cancer.

○———○ β-Glucuronidase activity (units/ml.).

●———● β-Glucuronidase activity (units/ml.) in urine containing blood.

patients were given (1) no treatment, (2) treatment with pure saccharo-1 : 4-lactone (1 g. six hourly), (3) no treatment, (4) treatment with a mixture of saccharo-1 : 4- and 3 : 6-lactones/2 : 3 (2·5 g. 6 hourly), (5) no treatment. In Case 4 the urine was contaminated with blood on the seventh day which accounts for the high result obtained.

Phenyl Glucosiduronic acid (I) | Glucosaccharo-1 : 4-lactone (II) | Glucosaccharic acid (III) | Glucosaccharo-3 : 6-lactone (IV)

TABLE VI.—*Patients Suffering from Cancer of the Bladder Treated with Pure Glucosaccharo*-1 : 4-*lactone* (1*g*. 4 *times daily*)

| Case No. | Sex | Age | Before treatment Vol./24 hr. | Before treatment Units/ml. | During treatment Vol./24 hr. | During treatment Units/ml. |
|---|---|---|---|---|---|---|
| 1 | M. | 53 | 1373 | 7·4 | 1527 | 0·84 |
| | | | 2013 | 5·2 | 1855 | 0·65 |
| | | | 1746 | 7·7 | 2120 | 0·51 |
| 2 | ,, | 62 | 2050 | 7·3 | 2105 | 0·88 |
| | | | 1760 | 6·3 | 2110 | 0·22 |
| 3 | F. | 76 | 2040 | 10·1 | 1980 | 0·60 |
| | | | 1660 | 7·7 | 1565 | 0·90 |
| | | | 2125 | 7·1 | — | — |
| 4 | M. | 66 | 2315 | 5·9 | 2110 | 0·88 |
| | | | 2740 | 6·6 | — | — |
| 5 | F. | — | 1470 | 12·6 | 2190 | 0·64 |
| | | | 1920 | 11·2 | 1695 | 0·44 |
| 6 | M. | 61 | 1980 | 3·0 | 2120 | 0·48 |
| | | | 2850 | 2·8 | 1870 | 0·32 |
| 7 | ,, | 82 | 1720 | 5·5 | 1560 | 0·22 |
| | | | 1600 | 4·1 | 1590 | 0·37 |

(4) The results obtained by treating patients with pure saccharo-1 : 4-lactone (0·5 g. 3 or 4 times daily) (Table VII) show a mean inhibition of 73 per cent and the effect on individual urine specimens of treating patients with 0·5 and 1 g. of saccharo-1 : 4-lactone 8 hourly is shown in Fig. 6.

FIG. 6.—The effect of oral administration of pure glucosaccharo-1 : 4-lactone on urinary β-glucuronidase activity in 3 patients with bladder cancer.
O———O β-Glucuronidase activity (units/ml.).

TABLE VII.—*Patients Suffering from Cancer of the Bladder Treated with Pure Glucosaccharo-*1 : 4-*lactone*

| Case No. | Sex | Age | Before treatment Vol./24 hr. | Before treatment Units/ml. | During treatment Vol./24 hr. | During treatment Units/ml. |
|---|---|---|---|---|---|---|
| (a) Dose 4 × 0·5 g./day)— | | | | | | |
| 1 | M. | 52 | 2720 | 1·1 | 2780 | 0·12 |
| | | | 2585 | 0·83 | 2740 | 0·12 |
| 2 | „ | 63 | 1415 | 1·5 | 1205 | 0·16 |
| | | | 1600 | 4·4 | — | — |
| 3 | F. | 43 | 1100 | 0·53 | 1550 | 0·06 |
| | | | 1700 | 0·38 | 1835 | 0·13 |
| (b) (Dose 3 × 0·5 g./day)— | | | | | | |
| 1 | M. | 43 | 1330 | 6·5 | 1515 | 3·8 |
| | | | 1205 | 6·9 | 1405 | 3·1 |
| 2 | „ | 55 | 2350 | 1·4 | 2140 | 0·17 |
| | | | 1560 | 1·5 | 2310 | 0·25 |
| 3 | „ | 68 | 2800 | 0·93 | 1830 | 0·10 |
| | | | 1590 | 1·0 | 2060 | 0·22 |
| 4 | „ | 71 | 2360 | 1·7 | 2290 | 0·37 |
| | | | 1510 | 1·9 | 2750 | 0·47 |

The results indicate that the urinary β-glucuronidase activity can be maintained at a low level by the administration of either the pure 1 : 4-lactone or the mixture of lactones. Patients are being treated with 1 g. saccharo-1 : 4-lactone *t.d.s.* for clinical trials.

### DISCUSSION

The urinary β-glucuronidase activities of patients who have been suffering from cancer of the bladder for some years are almost all raised above the corresponding value for normal subjects. The increased activity does not depend upon infection although, in a few cases infected by *B. coli*, this may play some part. The enzyme is stable up to pH 9·0 and the method of estimation allows urine of pH less than this value to be used. The results are in agreement with those obtained previously (Boyland *et al.*, 1955) using an earlier technique.

The urinary β-glucuronidase activity of patients with cancer of the kidney (hypernephroma) is low compared with that of bladder cancer patients, the average value being within the normal range. Fishman and his co-workers have shown that cancer cells have a high β-glucuronidase activity but the high urinary activity in cancer of the bladder cannot be due to the urine being in contact with malignant tissue as urine from patients with cancer of the kidney should also be rich in β-glucuronidase. This evidence further supports the suggestion (Boyland *et al.*, 1955) that the increased urinary enzyme concentration is probably derived from the blood, the amount of the enzyme depending both on the blood concentration and the degree to which the enzyme passes through the kidney. The relationship between the β-glucuronidase activities of serum and urine of patients with cancer of varying sites is being investigated further.

Patients suffering from cancer of the bladder have been treated with glucosaccharo-1 : 4-lactone (4 g. per day) which reduces the activity by 90 per cent. This inhibition of β-glucuronidase would be produced with a $5 \times 10^{-5}$ M saccharo-

1 : 4-lactone solution (Levvy, 1952) which is equivalent to about 10 mg. per litre. This indicates that only 0·5 per cent of the dose administered is excreted in the urine. The greater part (99·5 per cent) of the lactone is inactivated either by metabolic processes or reaction with water or other body constituents. This treatment reduced the urinary $\beta$-glucuronidase level to values below the average normal level and by this means it is hoped to decrease the amount of free carcinogens in the urine and thus reduce the incidence of new tumours in patients, who have had tumours removed. Twenty bladder cancer patients are at present under treatment with glucosaccharo-1 : 4-lactone, but this treatment has not been used long enough to allow any conclusions to be drawn as to the clinical benefit.

The authors realise that to treat patients who have had cancer of the bladder with compounds which will reduce the concentration of carcinogenic agents in the urine, is probably to act too late. Because of the long lag between the application of the carcinogenic stimulus and the occurrence of the cancer, the first stimulus had probably taken effect before treatment began. This is, however, not certain and the reduction of carcinogens in the urine may reduce the chance of tumours developing. The tumours may, however, be dependent on the continued presence of the carcinogen in the way that the growth of kidney tumours, induced in hamsters with stilboestrol is dependent on the continued presence of stilboestrol (Horning, 1956). If the tumours of the bladder were dependent on the urinary carcinogen then treatment with saccharo-1 : 4-lactone could prevent growth of established tumours.

Bladder cancer caused by aromatic amines among men in the chemical industry is probably due to metabolites of the aromatic amines being hydrolysed by $\beta$-glucuronidase in the urine to give aminophenols. Stringent precautions are now taken in the chemical industry to prevent exposure to the carcinogenic amines. In such factories, however, if accidents happen the carcinogenic effect of exposure of personnel should be reduced by administration of saccharo-1 : 4-lactone immediately following the exposure and for four or five days. In such cases the treatment should prevent the liberation of the carcinogen in the urine.

### SUMMARY

The *in vivo* effect of various derivatives of saccharic acid which are $\beta$-glucuronidase inhibitors has been investigated ; oral administration of 1 g. saccharo-1 : 4-lactone every 6 hours decreases the enzyme activity in the urine by 90 per cent.

Treatment with saccharolactone should therefore reduce the liberation of free carcinogenic aminophenols in urine. Such treatment is recommended for men accidentally exposed to carcinogenic aromatic amines and is being tried in prophylactic treatment of patients who have had bladder cancers removed or destroyed.

We are indebted to Mr. L. M. Miall of Messrs. Kemball Bishop and Co. Ltd., for his help in supplying many of the substances used in this work.

We should like to thank Mr. W. J. Gorrod and Mr. P. L. Grover for technical assistance. This investigation has been supported by grants to the Chester Beatty Research Institute (Institute of Cancer Research : Royal Cancer Hospital) from the British Empire Cancer Campaign, Jane Coffin Childs Memorial Fund for Medical

Research, the Anna Fuller Fund, and the National Cancer Institute of the National Institutes of Health, U.S. Public Health Service.

### REFERENCES

ALLEN, M., BOYLAND, E., DUKES, C. E., HORNING, E. S. AND WATSON, J. G.—(1957) *Brit. J. Cancer*, **11**, 212.
BONSER, G. M., BRADSHAW, L., CLAYSON, D. B. AND JULL, J. W.—(1956) *Ibid.*, **10**, 539.
BOYLAND, E., GASSON, J. E. AND WILLIAMS, D. C.—(1957) *Ibid.*, **11**, 120.
*Idem*, MANSON, D., SIMS, P. AND WILLIAMS, D. C.—(1956) *Biochem. J.*, **62**, 68.
*Idem*, WALLACE, D. M. AND WILLIAMS, D. C.—(1955) *Brit. J. Cancer*, **9**, 62.
*Idem* AND WILLIAMS, D. C.—(1954) *Biochem. J.*, **56**, xxix.—(1956) *Ibid.*, **64**, 578.
HORNING, E. S.—(1956) *Brit. J. Cancer*, **10**, 678.
KARUNAIRATNAM, M. C. AND LEVVY, G. A.—(1949) *Biochem. J.*, **44**, 599.
LEVVY, G. A.—(1952) *Ibid.*, **52**, 464.
MILLS, G. T. AND PAUL, J.—(1949) *Ibid.*, **44**, xxxiv.


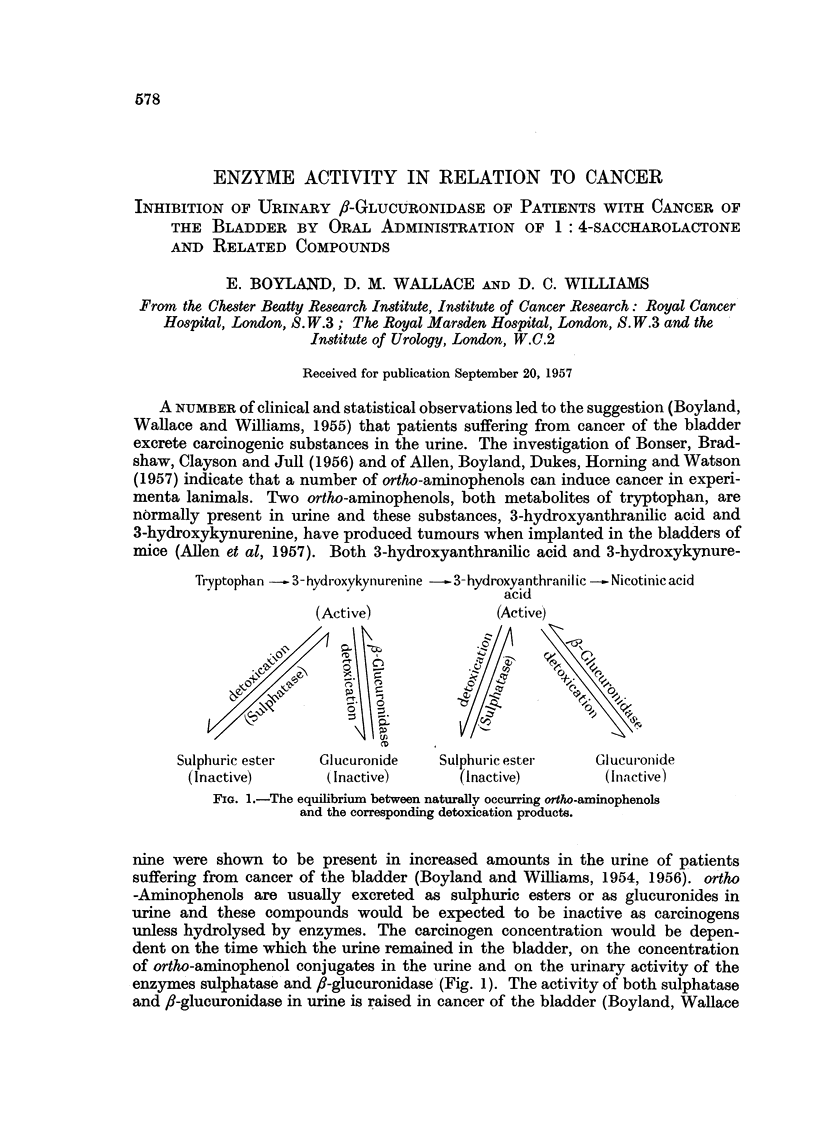

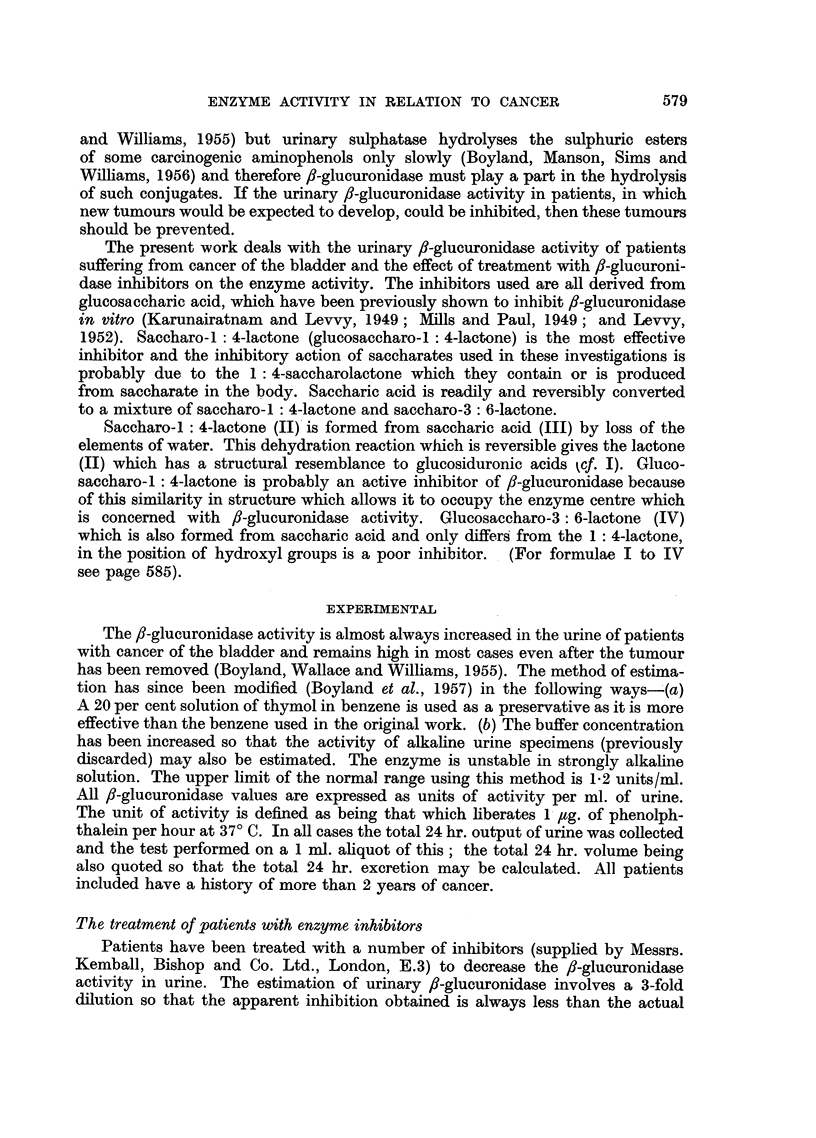

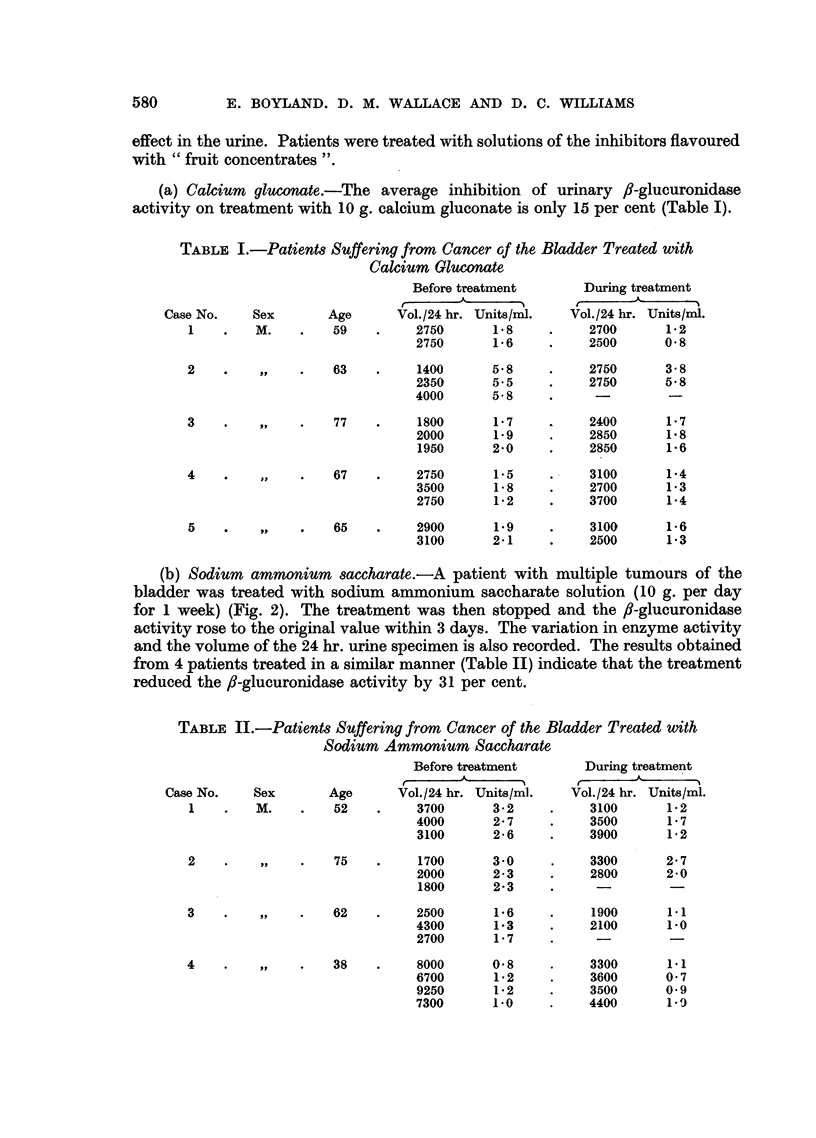

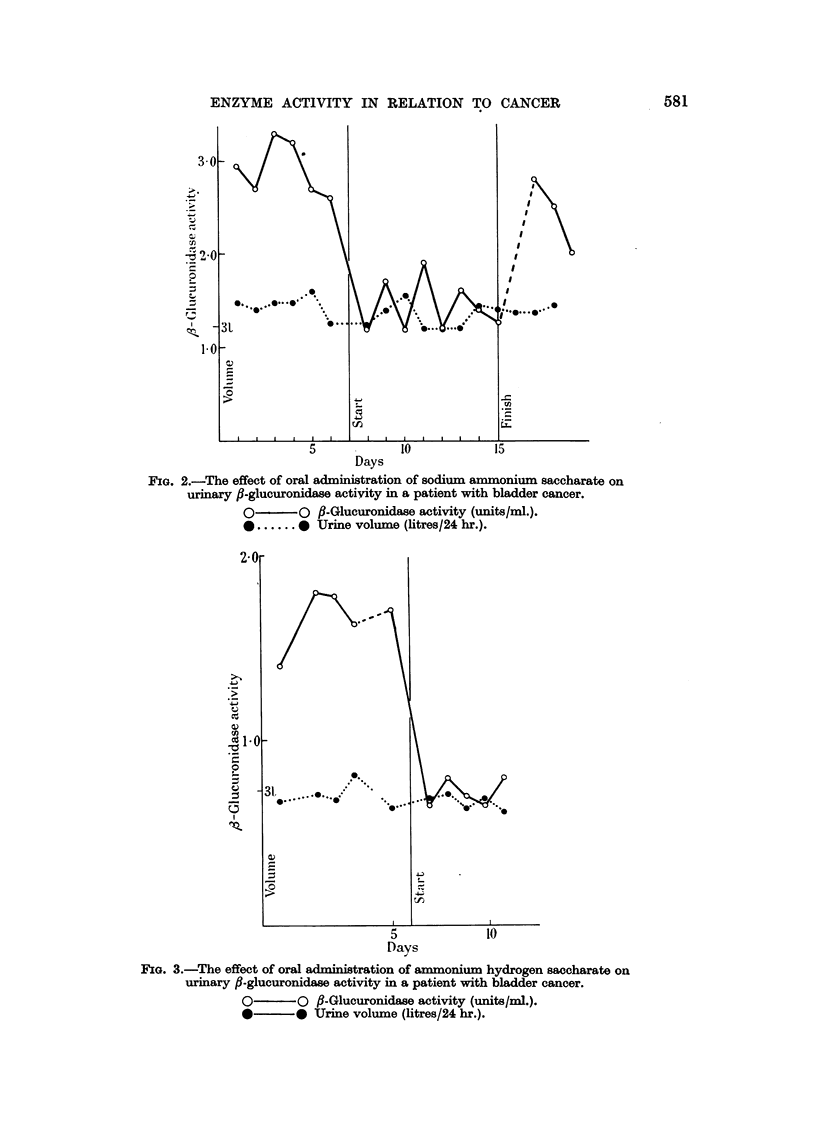

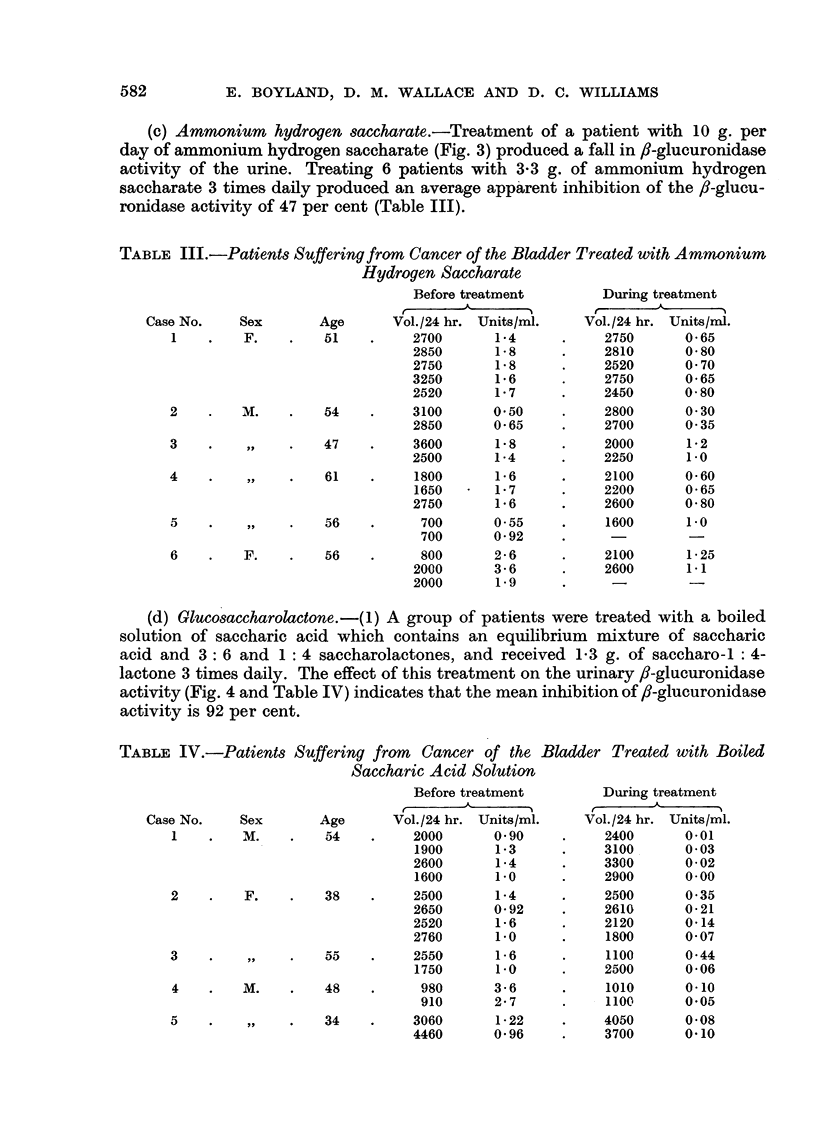

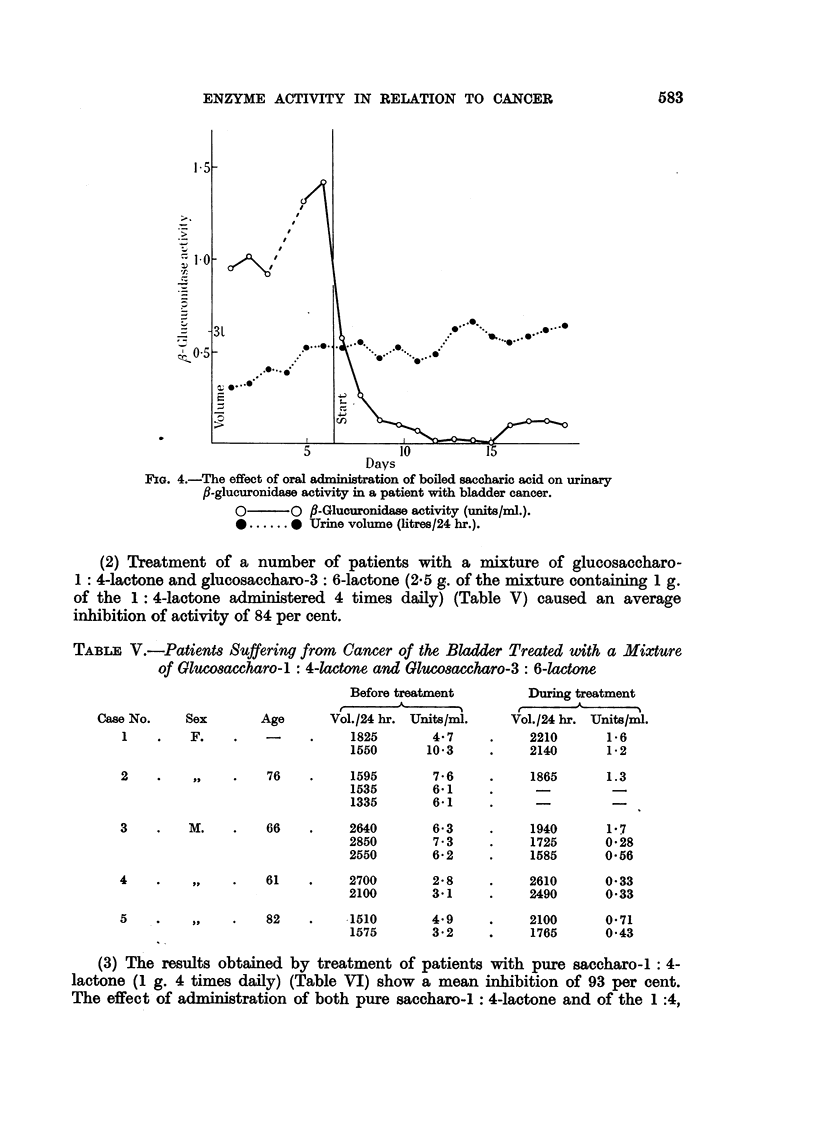

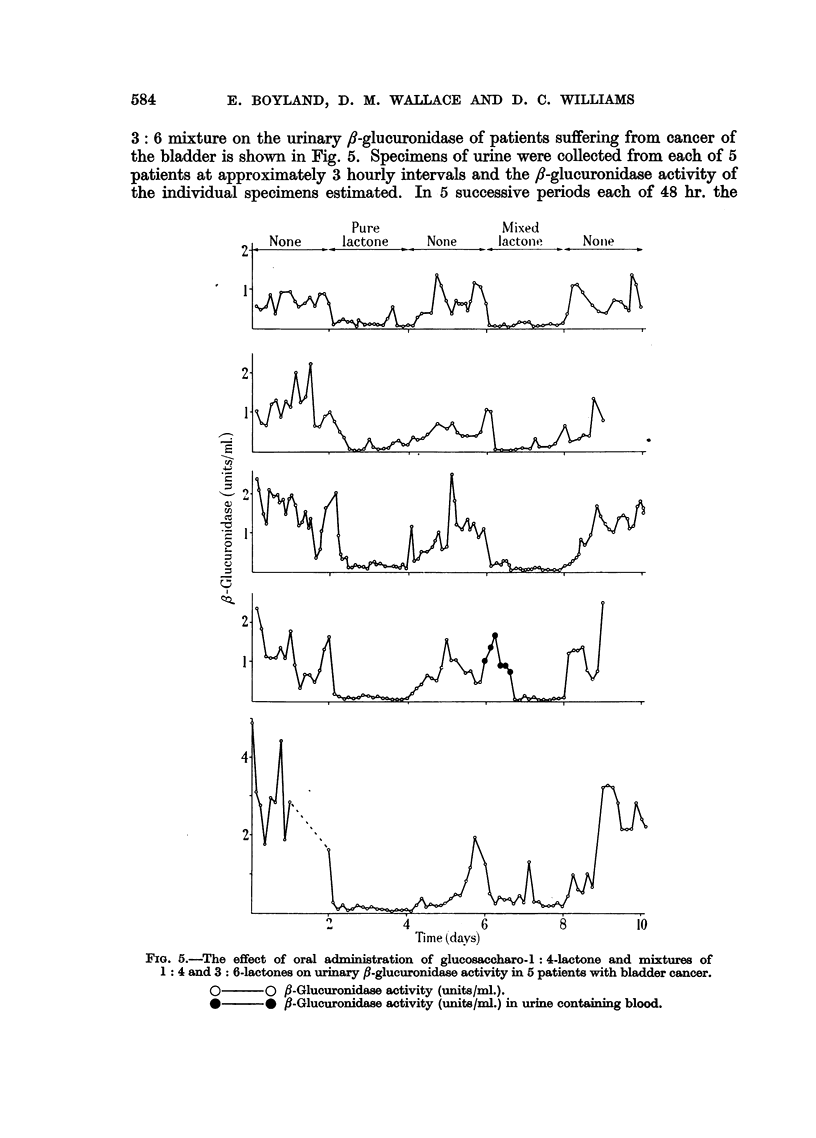

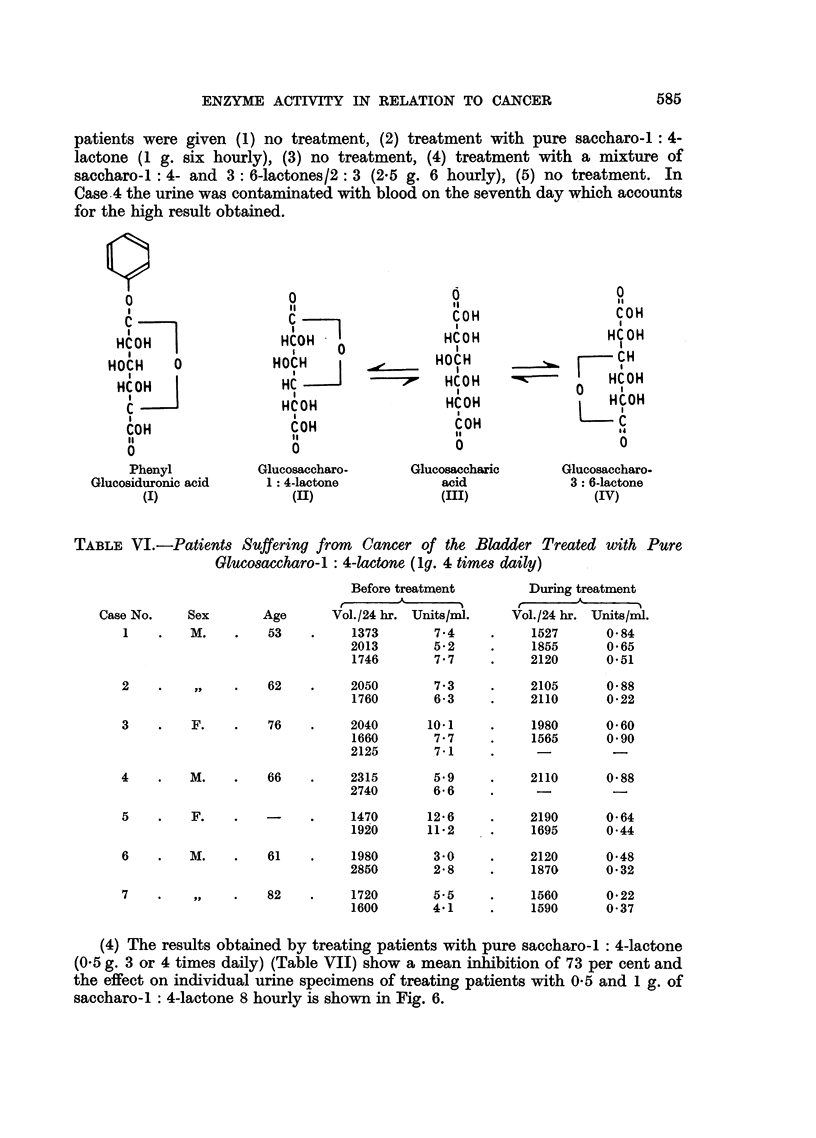

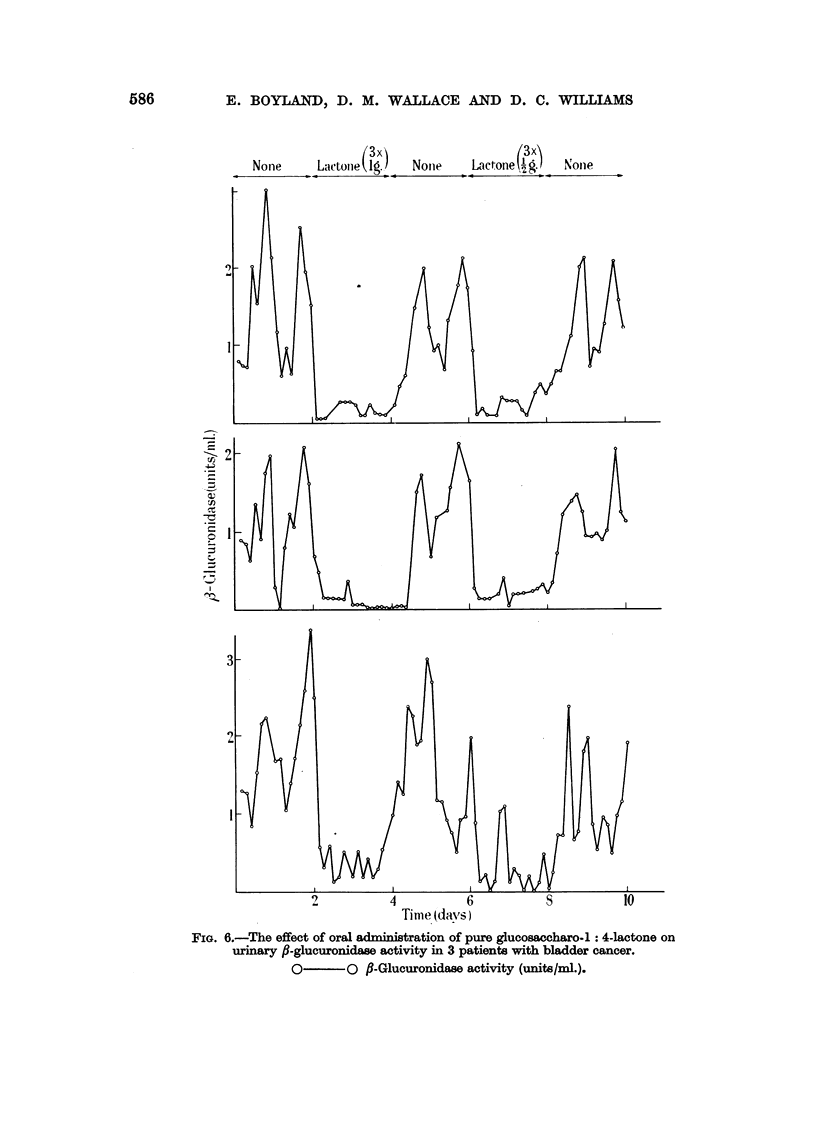

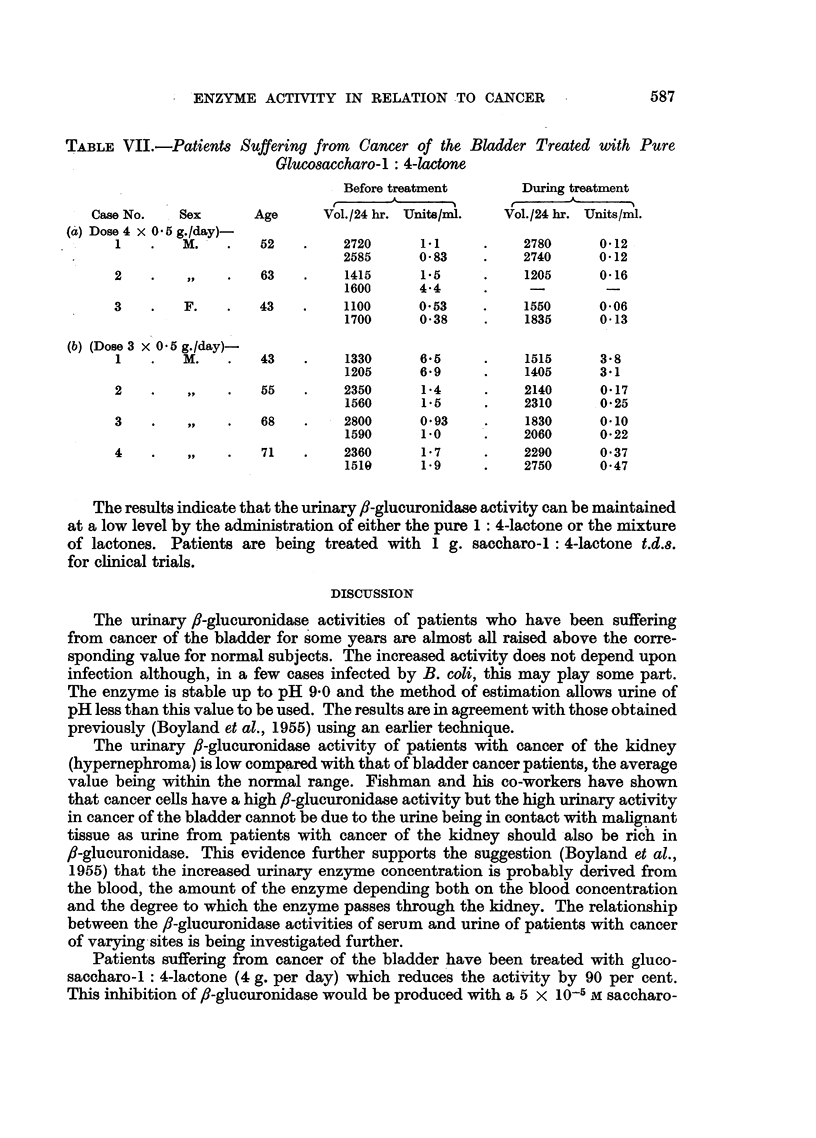

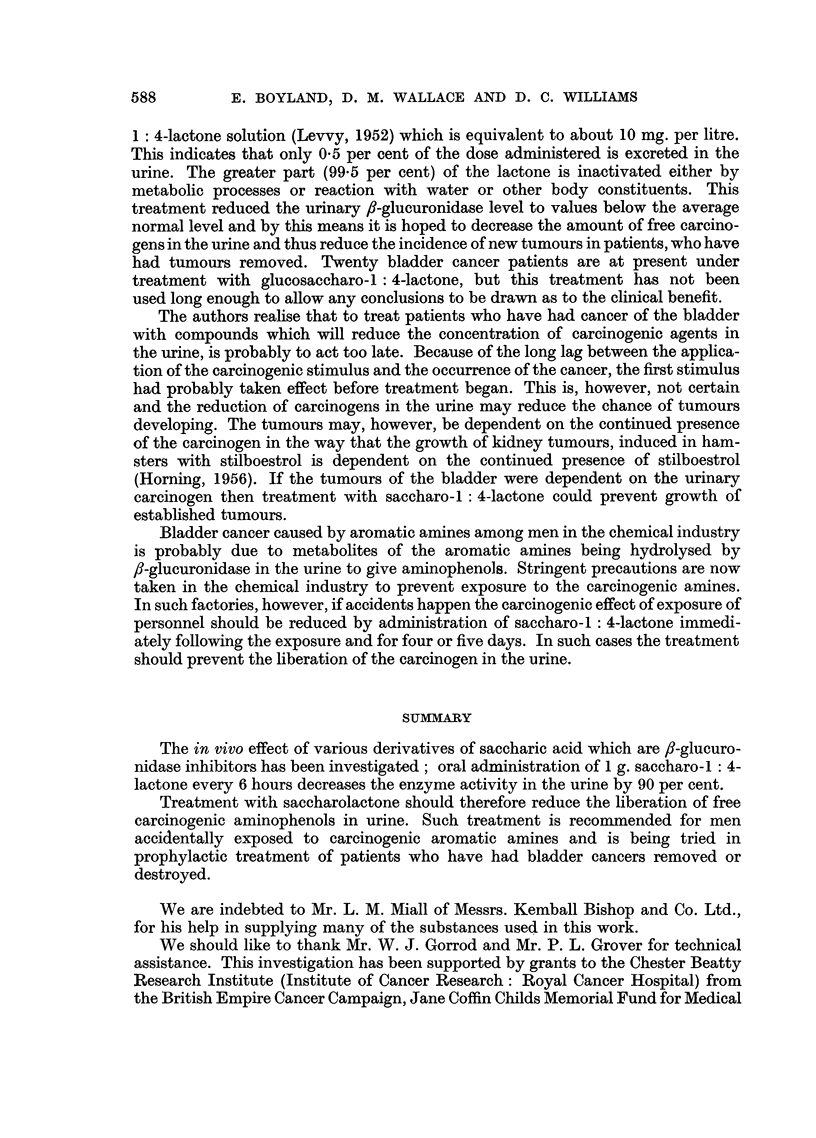

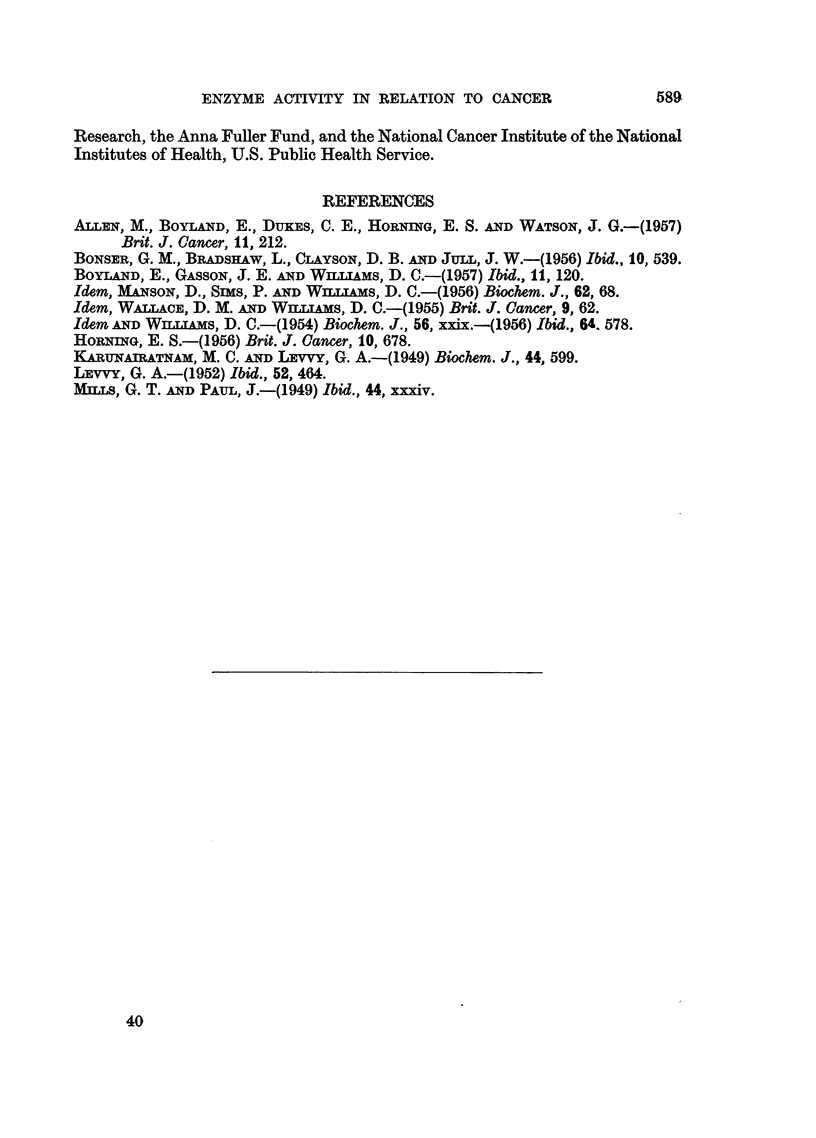


## References

[OCR_01046] ALLEN M. J., BOYLAND E., DUKES C. E., HORNING E. S., WATSON J. G. (1957). Cancer of the urinary bladder induced in mice with metabolites of aromatic amines and tryptophan.. Br J Cancer.

[OCR_01050] BONSER G. M., BRADSHAW L., CLAYSON D. B., JULL J. W. (1956). A further study of the carcinogenic properties of ortho hydroxy-amines and related compounds by bladder implantation in the mouse.. Br J Cancer.

[OCR_01051] BOYLAND E., GASSON J. E., WILLIAMS D. C. (1957). Enzyme activity in relation to cancer; the urinary beta-glucuronidase activity of patients suffering from malignant disease.. Br J Cancer.

[OCR_01057] HORNING E. S. (1956). Observations on hormone-dependent renal tumours in the golden hamster.. Br J Cancer.

[OCR_01059] Karunairatnam M. C., Levvy G. A. (1949). The inhibition of beta-glucuronidase by saccharic acid and the role of the enzyme in glucuronide synthesis.. Biochem J.

[OCR_01060] LEVVY G. A. (1952). The preparation and properties of beta-glucuronidase. IV. Inhibition by sugar acids and their lactones.. Biochem J.

